# Why do religious leaders observe costly prohibitions? Examining taboos on Mentawai shamans

**DOI:** 10.1017/ehs.2020.32

**Published:** 2020-06-11

**Authors:** Manvir Singh, Joseph Henrich

**Affiliations:** Department of Human Evolutionary Biology, Harvard University, Cambridge, MA 02138, USA

**Keywords:** Religion, shamanism, signalling, taboo

## Abstract

Religious leaders refrain from sex and food across human societies. Researchers argue that this avoidance influences people's perceptions of leaders’ underlying traits, but few, if any, quantitative data exist testing these claims. Here we show that shamans in a small-scale society observe costly prohibitions and that observers infer cooperativeness, religious belief, difference from normal humans and supernatural power from shamans’ adherence to special taboos. We investigated costly prohibitions on shamanic healers, known as *sikerei*, among the rainforest horticulturalist Mentawai people of Siberut Island. We found that shamans must observe permanent taboos on various animals, as well as prohibitions on sex and food during initiation and ceremonial healing. Using vignettes, we evaluated Mentawai participants’ inferences about taboo adherence, testing three different but not mutually exclusive hypotheses: cooperative costly signalling, credibility-enhancing displays and supernatural otherness. We found support for all three: Mentawai participants infer self-denying shamans to be (a) cooperative, (b) sincere believers in the religious rules and (c) dissimilar from normal humans and with greater supernatural powers. People's inferences about religious self-denial are multidimensional and consistent with several functional accounts.

## Graphical abstract

**Figure:**
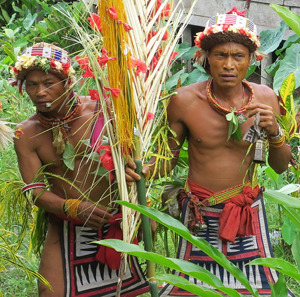

**Media summary:** We find that Mentawai shamans observe taboos on food and sex. Using a field experiment, we test three hypotheses as to why this is the case.

## Introduction

Prohibitions can be severe for religious leaders. Although laypeople often refrain from food or sex (Iannaccone, 1992; Olson & Perl, 2001), their avoidance tends to be mild compared to the normative self-denial imposed on leaders and practitioners. The Buddhist laity in China enjoys the freedom to ‘keep to a semi-vegetarian diet, or adopt a vegetarian diet for a limited period of time’ (Kieschnick, 2005, p. 186). In contrast, Buddhist ‘monks and nuns in China are expected to maintain a vegetarian diet, and as far one can tell, in general they take the prohibition seriously’ (Kieschnick, 2005, p. 186). Similarly, Jain laypeople must observe the five Lesser Vows, including avoiding being overly attached to their possessions (Dundas, 2002). Jain monks and nuns, meanwhile, abide by the much more severe Greater Vows, including abandoning their material possessions completely. And while their lay counterparts might occasionally abstain from sex, Aztec priests, Catholic priests and Daoist monks are (or were) required to be life-long celibates (Bell & Sobo, 2001; Herrou, 2012; Qirko, 2002). Importantly, leaders’ self-denial is not restricted to world religions and large-scale societies. Winkelman and White (1987) coded ethnographic information about trance practitioners (shamans) in a representative sample of human societies. Practitioners abstained from food, sex or social contact in 83% of societies coded (Singh, 2018).

Why should religious leaders observe such costly prohibitions, especially given that they can exploit their position to devise self-serving rules (Singh, Glowacki, & Wrangham, 2016; Singh, Wrangham, & Glowacki, 2017)? One proposal is that leaders’ self-denial promotes perceptions of underlying traits (Henrich, 2009; Norenzayan et al., 2016; Singh, 2018; Sosis & Alcorta, 2003). In this research, we tested three such hypotheses: cooperative costly signalling (Sosis & Alcorta, 2003), credibility-enhancing displays (Henrich, 2009) and supernatural otherness (Eliade, 1958; Mauss, 2001). These hypotheses posit, respectively, that observers infer cooperativeness, religious belief and supernatural power from normative self-denial. Crucially, these hypotheses are not mutually exclusive.

According to the cooperative costly signalling hypothesis, costly behaviours serve as reliable signals of an actor's cooperative intent (Bulbulia & Sosis, 2011; Gintis, Smith, & Bowles, 2001; Irons, 2001; Sosis & Alcorta, 2003). For example, costly behaviours might indicate that an individual is committed to cooperating with members of a particular group (Posner, 2000; Sosis, 2006) or that they believe in a religious system that includes cooperative doctrines (Henrich, 2009). Researchers have examined whether behaviours like possession and donating to charities act as costly signals of cooperativeness (Hall, Cohen, Meyer, Varley, & Brewer, 2015; Power, 2017a, b), but few if any quantitative studies have tested whether this hypothesis explains the ascetic practices of religious leaders.

Aside from broadcasting cooperativeness, self-denial may also indicate the sincerity or depth of one's belief. Developing this intuition, researchers have modelled how costly actions, termed credibility-enhancing displays (CREDs), coevolve culturally with certain beliefs (Henrich, 2009; Wildman & Sosis, 2011). CREDs function as cues to social learners that a cultural model (i.e. someone from whom social information is learned) genuinely subscribes to the beliefs he or she espouses. For example, consider a model who tells a learner that it is safe to consume some mushroom. In response to a learner's reasonable apprehension, the model can enhance their credibility by taking a bite of the mushroom – that is, by engaging in a behaviour that would be sensible only if the model genuinely believes what they espouse.

Researchers argue that cultural evolution may favour CREDs by ensuring the faithful transmission of religious beliefs and practices across generations. For instance, recent work suggests that costly religious displays may have developed to instill stronger beliefs in a powerful, moralizing god (Norenzayan et al., 2016). By this logic, religious systems may place prohibitions on religious leaders, often related to food, wealth and sex, because these taboos make them more effective as transmitters of the faith.

A growing body of research provides evidence for CREDs in different informational and behavioural domains (e.g. Kraft-Todd, Bollinger, Gillingham, Lamp, & Rand, 2018; Willard & Cingl, 2017). When betting on the validity of stories, subjects who witness others put down money are more likely to do the same (Willard, Henrich, & Norenzayan, 2016). Internet-users in the US who recall their caregivers engaging in behaviours such as religiously inspired charity are more likely to believe in God and hold that belief with greater confidence (Lanman & Buhrmester, 2017). Despite these advances, no work has tested whether a CREDs model explains the severe, religious restrictions of religious leaders.

Importantly, some behaviours can serve as both CREDs and cooperative costly signals (Bulbulia & Sosis, 2011), such as if a person demonstrates that they believe in a moralistic, punishing god. However, not all cooperative costly signals are CREDs – because a behaviour might signal cooperativeness through channels other than belief – and not all CREDs are cooperative costly signals – because, among other reasons, there are many beliefs that a person might have (such as that a mushroom is safe to eat) that do not make them more cooperative.

A third explanation for why practitioners observe costly prohibitions is what we refer to here as supernatural otherness. According to this hypothesis, observers infer that someone who self-denies is different from normal humans. This supposed difference makes it more conceivable that the self-denier has superhuman powers such as healing or divine contact (Eliade, 1958; Mauss, 2001). Researchers have not specified how or why observers should understand individuals who self-deny to be distinct from normal humans, but potential mechanisms include perceptions that they have strange preferences or unique cognitive abilities enabling their abstention or that they undergo a transformation as a result of the denial, such as becoming purer (Smith, 2007).

According to the supernatural otherness hypothesis, and at least some formulations of cooperative costly signalling, practitioners engage in self-denial for its downstream benefits. By signalling their prosocial disposition or by convincing others of their supernatural power, practitioners attract others’ trust and enhance perceptions of their singular abilities (Irons, 2001; Singh, 2018). Although a CREDs account is consistent with this self-interested use of asceticism, researchers studying CREDs have focused less on the benefits for individual practitioners and more on how normative self-denial enhances the transmission of prosocial religion (Norenzayan et al., 2016).

These three hypotheses posit that observers infer distinct traits from normative self-denial (Table 1), but they are not mutually exclusive. It might be the case, for instance, that observers infer cooperativeness, difference and supernatural power from leaders’ prohibitions but not religious belief. In such a case, we would find support for the cooperative costly signalling and supernatural otherness hypotheses while rejecting the CREDs hypothesis. Behaviours may serve many social functions rather than a single prototypical one.

**Table 1.** The distinguishing empirical patterns predicted by each of these three hypotheses for self-denying religious leaders; the table only includes those inferences wherein, were participants not to draw them, the corresponding hypothesis would be rejected

	Greater cooperativeness	Greater religious belief	Greater difference from normal humans	Greater supernatural power
Cooperative costly signalling	×			
CREDs (credibility-enhancing displays)		×		
Supernatural otherness			×	×

To test whether any or all of these hypotheses help explain why religious leaders observe costly prohibitions, we investigated taboos on shamans among the Mentawai of Siberut Island (Indonesia). Examining these dynamics among the Mentawai offers at least two advantages compared with research in industrialized populations with Abrahamic religions. First, as a belief system including, among other attributes, shamanism and an animist worldview (Loeb, 1929b, c; Schefold, 1988), Mentawai religion shares characteristics with the traditional religions of small-scale societies around the world, including those of hunter–gatherers (Peoples, Duda, & Marlowe, 2016; Singh, 2018). Studying Mentawai religion thus permits generalizations to a diversity of contexts, most of which are less relevant when examining the centralized religions of complex societies (Boyer, 2019; Boyer & Baumard, 2016). Second, religious systems include many practices and beliefs, some of them recent variations owing to cultural drift, others more functionally important and stable over time (see Currie & Mace, 2014; Rogers & Ehrlich, 2008). The unique arrangement of Siberut Island, which contains many cultural regions that differ slightly in their practices and beliefs, allows us to identify those taboos on shamans that are shared across cultural regions and thus more likely to be functionally important.

### Mentawai and *Sikerei*

**Figure 1.**
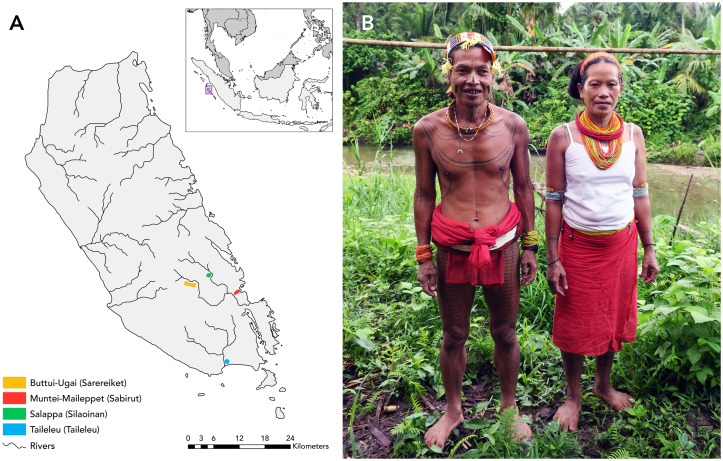
(a) Siberut Island, the largest island of the Mentawai Archipelago (Indonesia). Coloured dots represent different study sites; the legend specifies the villages surveyed with the cultural region in parentheses. Indonesia is coloured light grey in the inset, while other countries are in dark grey. (b) A Mentawai shaman and his wife.

The Mentawai of Siberut Island are forest-dwelling sago-horticulturalists who live in river valleys separated by hilly expanses of forest (Tulius, 2012) (Figure 1a). At least 11 major valleys cover the island, each hosting a set of communities who speak their own dialect and decorate themselves with unique tattoo motifs. Throughout the rest of this paper, we refer to the set of communities who reside in the same valley and share a dialect as a cultural region.

Missionaries and government programmes have transformed the religious lives of people living elsewhere in the Mentawai Archipelago, but dense tracts of forests have hindered these efforts on Siberut (Schefold, 1988). This, in combination with struggles by Mentawai clans to resist these programmes, has allowed the traditional religious system to survive relatively intact in the interior of Siberut, bolstered by a strong shamanic institution. Nevertheless, tourism, the spread of Islam, settlement agendas by the government and the expansion of formal education are rapidly transforming Mentawai social and cultural life (Delfi, 2013, 2017; Hammons, 2010), making ethnographic investigations invaluable.

Mentawai shamans are a class of men believed to possess the unique ability to see otherwise invisible spirits (Loeb, 1929c). These spirits include the ghosts of ancestors, deities that cause sickness (e.g. *Sikameinan*, a water-dwelling spirit, sometimes described as a crocodile, who punishes stinginess (Singh, Kaptchuk, & Henrich, 2019)) and human souls, whose departure from the body manifests as illness. As healers, shamans are experts in herbal medicine and the special songs used for communicating with various spirits. In some cultural regions, shamans are marked by their continued use of the loincloth and their full-body tattoos (Figure 1b).

Shamans and their wives are both known as *sikerei*; other individuals are referred to as *simata* (uncooked, unripe). Because, with few possible exceptions, only male *sikerei* are believed to see spirits and are invited to heal illness during healing ceremonies – and because the term is commonly used to describe those individuals who provide these services – we use *sikerei* to refer to male *sikerei* unless otherwise specified.

*Sikerei* treat illness in *pabetei*, healing ceremonies that last from one day to a week. Families of sick people invite one to six shamans to administer treatments, which can include providing herbs, sweeping away evil spirits, beckoning a sick person's soul, and summoning the water spirit *Sikameinan* and removing it from the house. Shamans enter trance during a special nighttime treatment, known as *lajok simagre*, during which several shamans dance and summon beneficent spirits, some of whom possess the practitioners.

Shamans benefit from healing patients through gifts of meat. As a part of healing ceremonies, patients and their families must sacrifice pigs and chickens (Singh et al., 2019). These are shared with family members as well as with the attending shamans, who consume the meat at the ceremony and are given portions to take home. Different interventions require different sacrifices, and many people informally refer to the sacrificed animals as a form of payment (Singh et al., 2019).

**Figure 2.**
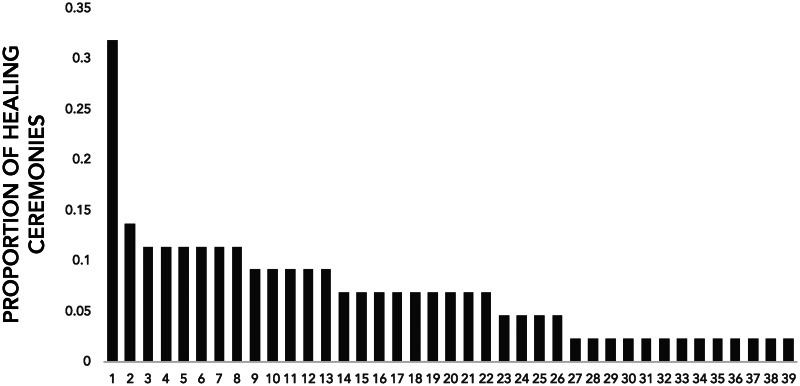
The frequency with which 39 different shamans were called to heal patients in a sample of 44 ceremonies. Each number on the horizontal axis refers to a different shaman.

Not all shamans are equally successful. Figure 2 shows how often different shamans were called to heal patients in a sample of 44 healing ceremonies occurring in two villages in interior Siberut (see Singh et al., 2019 for details on the dataset). Notably, a single shaman was called for 14 ceremonies (32%); the next most successful shaman was called for 6 (14%). The median number of ceremonies in which a shaman healed a patient was 3; the mode was 1. That shamans compete for profitable healing opportunities suggests that they should benefit from promoting perceptions of prosociality, credibility and supernatural power.

A man hoping to become a shaman can start by erecting and inhabiting a small house in the forest (*pulaeat*), observing taboos while dedicating himself to raising chickens and pigs. After days or weeks there, the novice finds a shaman-guru (*sipauma*), who typically demands pigs, durian trees, coconut trees and sago. In return, the guru teaches the novice the herbal remedies and songs required for healing while treating the trainee's eyes to help him see spirits. Some initiates do not move to a forest house; instead, their training begins with a severe, untreatable illness, perceived by others as a sign that a person must become a shaman.

The *sikerei* must constantly observe taboos, or *keikei* (Loeb, 1929b; Schefold, 1988). As with shamans around the world (Eliade, 1964; Narby & Huxley, 2001), the Mentawai *sikerei* must abstain from sex and various food items during initiation and ceremonial periods, in addition to permanently refraining from several hunted animals. The severity of sex taboos in particular is captured in the frequent remark that, in becoming a *sikerei*, one's wife becomes one's sister.

We used costly prohibitions on Mentawai shamans (*keikei sikerei*) to evaluate whether and how observing religious taboos promotes perceptions of cooperativeness, credibility and supernatural power. We first documented those taboos that apply to shamans and investigated the costliness of a subset of those prohibitions. We then used vignettes to probe participants’ inferences about self-denying shamans and test the three hypotheses reviewed earlier.

## Study 1: what are the taboos on shamans?

We first documented the activities and food items that are tabooed to shamans during initiation, during healing ceremonies and permanently. By examining which prohibitions are shared across river valleys, we identified the taboos that are most resistant to change and which thus seem most functionally important (Currie & Mace, 2014).

### Methods

We interviewed 88 participants about taboos on shamans across four cultural regions of southern Siberut (see Figure 1a; Sabirut: *n* = 20; Sarereiket: *n* = 27; Silaoinan: *n* = 21; Taileleu: *n* = 20), asking about temporary taboos during initiation and healing as well as permanent taboos that apply through a shaman's lifetime. One participant was excluded from the permanent dietary taboo condition because of admitted ignorance. All participants provided informed consent before the study. The Harvard University Committee on the Use of Human Subjects approved this study and all others described in this paper.

We collected initiation and healing taboos using free-lists. We collected permanent dietary taboos, in contrast, using a checklist of 14 items. We developed the checklist after administering pilot interviews in three cultural regions (Sareireket, Sabirut and Simatalu). Thirteen of the 14 items in the checklist were those mentioned by more than one respondent during pilot interviews. The fourteenth (Mentawai langur, *Presbytis potenziani*) is commonly said to be freely consumed by shamans and was included to both confirm participant comprehension and to discourage participants from assuming that all items on the list were tabooed.

We excluded instances when participants specified that an item on the checklist was tabooed to shamans only during special periods. If a respondent mentioned that an item was permanently tabooed to a shaman but also temporarily tabooed during initiation and healing, we only included it as a permanent prohibition. If a participant listed a permanent dietary taboo but then specified that it only applied during healing ceremonies, we included it only as a periodic taboo during healing. We categorized taboos at two levels, first grouping similar responses and then aggregating those taboos within superordinate categories, such as taboos pertaining to sex, eating and grooming.

**Figure 3.**
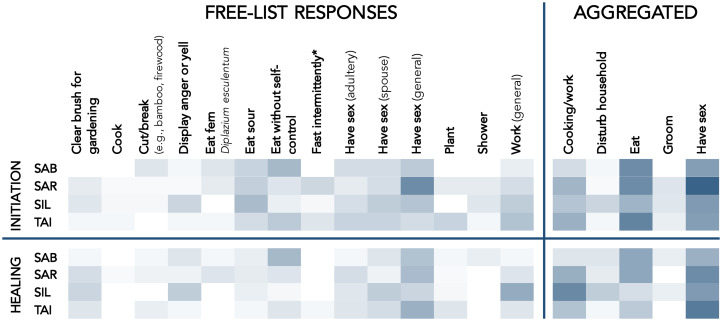
Prohibitions on shamans during initiation and healing ceremonies, according to free-lists by respondents in four cultural regions of southern Siberut. Rows correspond to responses from the regions of Sabirut (SAB), Sarereiket (SAR), Silaoinan (SIL) and Taileleu (TAI). White cells occur when no participants in a cultural region reported a taboo; dark blue cells occur when all participants reported a taboo; transitional shades denote intermediate frequencies. The free-list response columns only include those taboos that were reported in at least three cultural regions for at least one domain. ‘Fast intermittently’ is labelled with an asterisk because it is a prescription rather than a prohibition. The five aggregated columns refer to super-ordinate categories that contain the responses on the left and others; for example, ‘Cooking/work’ includes ‘Clear brush for gardening’, ‘Cut/break’, ‘Plant’, ‘Work (general)’ and other work-related prohibitions that were reported in low frequencies. Raw frequencies appear in Supplementary Table S1.

We did not use inferential analyses on the free-list data; instead we present the raw frequencies of commonly cited taboos (see Figure 3 and Supplementary Table S1). The checklist data, on the other hand, was analysed with cultural consensus analyses using the ‘AnthroTools’ package in R (Purzycki & Jamieson-Lane, 2017).

### Results: taboos during healing and initiation

Figure 3 shows those items that respondents mentioned in at least three cultural regions for at least one of the two categories of prohibition. Participants reported 54 taboos on shamans during initiation, 13 of which were mentioned in at least three cultural regions. Seven prohibitions appeared across all of the sites: eating without self-control, eating sour foods, committing adultery, having sex with one's spouse, having sex with anyone and doing any kind of work (including hunting, repairing a house, or tending to one's gardens). To eat with self-control means to eat only when sitting down in a house with others, ideally when the food being served was prepared at an earlier time. To eat without self-control, in contrast, is to eat while walking or casually sitting, or to eat food that has been foraged and immediately prepared, like freshwater fish or taro leaves.

Taboos during healing ceremonies are similar to those observed during initiation. Participants mentioned 52 prescriptions that apply to shamans during healing. No taboos were mentioned in only three cultural regions; nine were mentioned in all study regions. As with taboos on shamans during initiation, taboos during healing ceremonies center on work, food and sex. Figure 3 displays the frequencies with which participants listed different prohibitions along with aggregated frequencies (see Supplementary Table S1).

### Results: permanent dietary taboos

**Figure 4.**
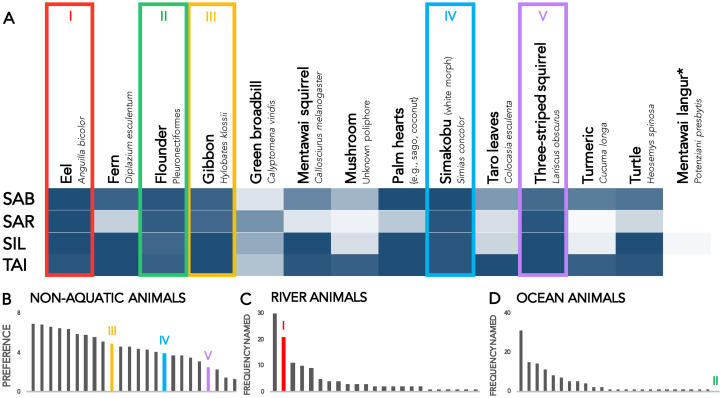
Permanent dietary taboos on shamans (a) and individuals’ preferences for those food items (b–d). (a) Participants in four cultural regions of southern Siberut (Sabirut, Sarereiket, Silaoinan and Taileleu) answered ‘yes’ or ‘no’ to whether 14 food items are permanently tabooed to shamans. White cells occur when no participant reported that an item was prohibited; dark blue cells occur when all participants reported a prohibition; transitional shades denote intermediate frequencies. Cultural consensus analyses identified five food items as being tabooed across all four regions, indicated in colored boxes. The Mentawai langur was included as a control because it is freely and commonly consumed by shamans. (b–d) Because of methodological limitations, different tasks were administered to measure how the prohibited foods ranked in people's dietary preferences. (b) Respondents’ preferences for 24 foraged animals, including the three non-aquatic species consistently prohibited to shamans [III, gibbon; IV, simakobu monkey (white morph); V, three-striped squirrel]. (c, d) The number of times different river (c) and ocean (d) animals were named as the most preferred and frequently consumed items; the items tabooed to shamans were mentioned second most frequently (I, eel) and not at all (II, flounder). Raw frequencies appear in Supplementary Table S2.

Supplementary Table S2 shows the raw frequencies with which respondents reported different permanent dietary taboos on shamans. Using cultural consensus analyses, we determined that five animal species are tabooed in all four cultural regions: eels (*Anguilla bicolor*), flounders (Pleuronectiformes), gibbons (*Hylobates klossii*), the white morph of the simakobu monkey (*Simias concolor*) and three-striped squirrels (*Lariscus obscurus*) (see Figure 4 and Supplementary Tables S3 and S4). Confirming respondents’ honesty and comprehension, only one participant of 87 replied that Mentawai langurs are tabooed, and they specified that this was a special case that required unique circumstances.

## Study 2: how costly are shamans’ dietary taboos?

We found that shamans across southern Siberut observe periodic prohibitions on sex and unconstrained eating. They are also permanently prohibited from five foraged food items. The periodic taboos, especially those on sex, appear decisively costly, but the costliness of the permanent dietary taboos is more unclear. We thus used ranking tasks to establish the relative costs of abstaining from eels, flounders, gibbons, white simakobu monkeys and three-striped squirrels.

### Methods

To determine whether and how costly permanent dietary taboos are for shamans, we conducted two tasks probing Mentawai participants’ dietary preferences. First, we investigated the costs of giving up non-aquatic, tabooed animals. We assembled as exhaustive a list as possible of Mentawai wildlife by consulting the appendix of a conservation plan printed by the Indonesian Ministry of Forestry (PHPA, 1995), collected photographs of each species mentioned in the report and convened with focus groups of Mentawai participants to determine the edibility and local name of each animal. The compiled list excluded all aquatic species, representing only *iba sibara ka leleu* (meat of the jungle). After incorporating edible insects and molluscs, we concluded with a list of 77 consumed, non-aquatic species (74 species excluding the items commonly tabooed to shamans).

To determine how the three tabooed, non-aquatic food items ranked in comparison with other non-aquatic, foraged animals, we presented non-shamans (*n* = 40) with photographs of 24 animals, 21 randomly drawn from the list of 74 animals alongside the three tabooed species found on land [*Hylobates klossii*, *Simias concolor* (white), *Lariscus obscurus*; see Supplementary Table S5 for a list of the 24 animals]. All participants gave informed consent. The photographs were presented randomly in a grid of four photographs by six photographs. Participants divided the 24 items into two groups of 12, one including animals they would never eat again and one including animals they would continue to eat. They subdivided these groups again, and so on and so forth, until they produced eight piles, producing a ranking of foraged items from ‘most willing to give up’ (ranking = 1) to ‘least willing to give up’ (ranking = 8). We calculated each item's mean ranking.

We targeted willingness-to-give-up because this integrates people's preference for an animal with its availability. For example, denying oneself a high-quality animal that is rarely encountered is less costly than rejecting an item that is of slightly lower quality but frequently encountered. Willingness-to-give-up captures this asymmetry, and participants appeared to consider both their preference for an item and its availability when making their decisions.

Because we could not obtain a list of consumed, aquatic species with accompanying photographs, we administered a different task to determine the costs of renouncing eels and flounders. We asked the same subjects to name six aquatic, foraged animals, selecting three from the river and three from the ocean. We specifically asked participants to consider both the frequency with which they ate those items and their preference for those animals in making their selections. We identified synonyms and determined each item's ranking by counting the number of times it was named.

### Results

The items vary considerably in people's self-reported willingness to give them up (Figure 4b–d). Among the 24 foraged land animals, items tabooed to shamans ranked as tenth (*Hylobates klossii*), sixteenth [*Simias concolor* (white)] and twenty-first (*Lariscus obscurus*) in people's dietary preferences (see mean rankings and standard deviations in Supplementary Table S5).

In naming the river species that they enjoyed the most and consumed most frequently, participants listed the item tabooed to shamans, *Anguilla bicolor*, second, preferring it to all freshwater species aside from shrimp. In marked contrast, no participant mentioned flounders when naming favoured saltwater species.

## Study 3: what do observers infer about self-denying shamans?

We have established that Mentawai shamans observe costly periodic prohibitions, as well as permanent dietary prohibitions with more ambiguous or varied costs. We therefore conducted an experiment in the field to probe whether self-denial by shamans promotes any dimension of religious credibility, specifically testing the predictions of the cooperative costly signalling, CREDs and supernatural otherness hypotheses.

### Methods: participants and procedure

Participants (*n* = 96) were opportunistically recruited in two villages in the interior of Siberut Island. All participants gave informed consent.

We presented each participant with two shamans introduced as pretend characters. We counterbalanced the characters’ images and background details, including how many children they have, whether they are knowledgeable about making canoes and whether they have lots of gardens. We also randomized the category of self-denial (food/sex). In the following example, the first character (Aman Dong Dong) refrains from foraged food items; the counter-balanced information has been labelled and distinguished from the treatment information:

*Self-denying character*
Aman Dong Dong, here, is a shaman. He follows all of the shaman taboos.
He has two children; he is knowledgeable in making canoes. [*Counter-balanced text*]
He does not eat pangolins, Pagai Island macaques, and flying foxes. [*Treatment text*]

*Control character*
Aman Paule, here, is also a shaman. He follows all of the shaman taboos.
He has three children; he has a lot of gardens. [*Counter-balanced text*]
He does not like to eat chili; he likes to eat cassava leaves. [*Treatment text*]

The treatment text in the sex condition was ‘Every day he does not sleep with [ = have sex with] his wife’. The control text in the sex condition was the same (‘He does not like to eat chili; he likes to eat cassava leaves’).

We specified that the shaman abstains from eating pangolins, Pagai Island macaques and flying foxes, because these animals, like two items tabooed to shamans (gibbons, white simakobu monkeys), constitute a class of hunted animals afforded special reverence, known as *matei keccak* (dead souls).

We then asked participants two comprehension questions that also served to prime the relevant information: (a) ‘Who does not eat flying foxes?’ (food condition) or ‘Who does not sleep with their wife every day?’ (sex condition); and (b) ‘Who does not like to eat chili?’ If a participant failed, we re-read the character descriptions. We then asked participants questions about the characters’ belief, cooperativeness, power and difference from other humans (see Supplementary Materials for the full list of questions). The questions were administered in one of four randomized orders. We again asked the comprehension questions at the end of the experiment to verify that participants remembered which character exhibited which traits. Supplementary Table S6 and Supplementary Figures S1 and S2 present summaries of the raw data.

### Methods: analysis

We conducted the task with 96 participants. To ensure the quality of the data reported, we only analysed the responses of respondents who passed a series of comprehension and attention checks. We used an especially conservative set of inclusion criteria in an attempt to remove any individuals who may have misunderstood the experiment. First, we excluded subjects who failed the final comprehension check (*n* = 12). Second, we excluded participants whose answers contradicted themselves for at least three of four pairs of questions targeting the same inference (questions beli1 and beli3; coop1 and coop2; powe1 and powe2; diff1 and diff2 in the Supplementary Materials; *n* = 11). Third, we excluded participants who, in their responses, alternated between the characters for at least 13 of the 14 questions (*n* = 9) (e.g. naming the first character, then the second, then the first, and so on). Twenty-six participants failed at least one check (six failed more than one). Two more participants were removed from analyses for experimenter error, yielding a final sample size of 68 respondents. The analyses produce largely similar results when including excluded participants, although the effect sizes are smaller (Supplementary Table S10).

We conducted all statistical analyses in R (R Core Team, 2015). The code used for analyses is available on the OSF project website (https://osf.io/3mbkz).

We used exploratory factor analysis to test whether each of the four set of questions were unidimensional (see Supplementary Table S7 for details; Supplementary Table S8 shows the results of a factor analysis conducted across all questions). Questions about belief and power were each unidimensional. Questions about cooperativeness and difference were not. Two questions about cooperativeness (coop3 and trus1) and one question about difference (diff1) did not load with the other questions. We thus removed those questions from subsequent analyses. Supplementary Table S9 shows other measures of internal reliability and unidimensionality for all sets of questions both before and after removing questions (values after removing questions: for belief, cooperativeness, and power, Cronbach's alpha > 0.75; for difference, Cronbach's alpha = 0.55; for all four sets, 0.31 ≥ average inter-item correlation ≥ 0.58; for all four sets, unidimensionality criterion = 1). Importantly, removing questions had nearly no effect on the later results (Supplementary Table S11).

Using the ‘glmer’ function of the ‘lme4’ package (Bates, Maechler, Bolker, & Walker, 2015), we ran a mixed effects logistic regression to model the likelihood that a participant selects the self-denying character as a function of a set of predictors. Our main predictor of interest was a factor whose levels correspond with the different kinds of inferences (belief, cooperativeness, difference, power). This allowed us to ask what the likelihood is that participants choose the self-denying shaman given that they are asked about, for instance, belief (or cooperativeness, difference, or power). We included sex, counterbalanced information and category of self-denial (food or sex) as covariates and a random effect for each participant ID. We used the ‘effects’ package (Fox & Weisberg, 2019) to produce average probabilities and the ‘emmeans’ package (Lenth, 2019) to test (a) whether the probabilities differed from each other, adjusting for multiple comparisons using Holm–Bonferonni, and (b) whether the category of self-denial (food or sex) had an effect on trait inferences.

### Results

**Figure 5.**
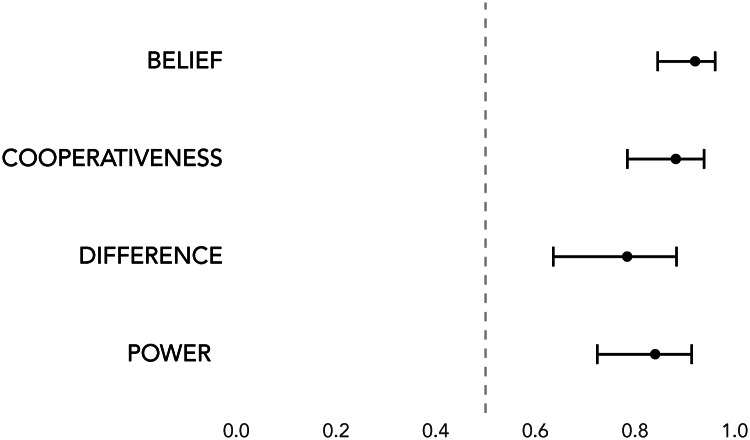
Estimated probabilities that a participant reports a self-denying shaman as having a particular trait. A probability of 1 indicates that participants always infer self-denying shamans to exhibit that trait, whereas a probability of 0 indicates that participants never infer self-denying shamans to exhibit that trait. A probability of 0.5, marked with a dotted line, indicates chance. Error bars represent 95% confidence intervals.

Supplementary Table S12 displays the results of the logistic regression. Figure 5 displays the mean estimated probabilities that a participant selects the self-denying character for questions about each of the four traits. Respondents are more likely to regard the self-denying shaman as a stronger believer in Mentawai religious beliefs [average estimated probability (*Pr*) = 0.92; 95% CI = (0.85, 0.96)], more cooperative [*Pr* = 0.88; 95% CI = (0.78, 0.94)], more different from normal humans [*Pr* = 0.78; 95% CI = (0.64, 0.88)], and more supernaturally powerful [*Pr* = 0.84; 95% CI = (0.72, 0.91)].

We conducted pairwise comparisons among all coefficients to test for differences. We found that the odds that a participant selects a self-denying shaman for a question about belief were 3.19-times the odds that they chose the tabooed shaman for a question about difference (SE = 1.08; *z*-ratio = 3.42; *p* < 0.01). We also a found a marginally significant difference between the odds that a participant selected a self-denying shaman for a question about belief as compared with the odds they chose the self-denier for a question about power (odds-ratio = 2.20; SE = 0.68; *z*-ratio = 2.54; *p* = 0.055). Otherwise, there were no significant differences among the coefficients (see Supplementary Table S13).

We found no effect for the category of self-denial (odds ratio = 0.399; SE = 0.242; *p* = 0.13). In other words, respondents made similar inferences about shamans who refrained from sex as they did about shamans who refrained from eating various food items.

## Discussion

Mentawai taboos impose enduring costs on shamans, such as by barring them from having sex during healing ceremonies and permanently forbidding them from consuming valued items. We tested three hypotheses for why shamans observe these taboos: cooperative costly signalling, CREDs and supernatural otherness. Our field experiment found evidence for all three hypotheses. Participants infer shamans who abstain from food and sex to be more cooperative, sincere in their belief, supernaturally powerful and psychologically and physically dissimilar. The strongest effects were on perceived belief.

This research is significant for at least four reasons. First, it represents a quantitative examination of why religious leaders engage in costly self-denial. Although evolutionary and psychological researchers have studied practices like possession, ritual scarring and church attendance (Hall et al., 2015; Power, 2017a; Sosis, Kress, & Boster, 2007), and although they commonly posit functional hypotheses for leaders’ celibacy and asceticism (Henrich, 2009; Norenzayan et al., 2016; Singh, 2018; Sosis & Alcorta, 2003), little to no quantitative research has tested why leaders observe these prohibitions.

Second, this study provides the first experimental evidence that costly behaviours can simultaneously garner perceptions of supernatural power and difference from normal humans. This is consistent with wisdoms about the metamorphic nature of pain and hardship (Glucklich, 2001; Hoffman & Trawalter, 2016; McCullough & Willoughby, 2009), but it suggests that, whether or not pain and denial *actually* change a person, psychological mechanisms predispose people to interpret those behaviours as transformative.

Third, this research advances our understanding of the origins of institutionalized leadership in human societies. Religious authority often translates into leadership roles beyond the supernatural, such as in providing social security, organizing economic activity or arbitrating conflict (Singh, 2018; Soler, 2016). This research identifies self-denial as a potential mechanism by which shamans and other practitioners maintain their authority, even in small-scale societies.

Finally, this research is significant because it demonstrates that people's inferences about costly religious behaviour are multidimensional and potentially compatible with several evolutionary functions. Evolutionary and psychological scientists have documented a large body of evidence in support of the hypothesis that costly religious behaviour is a signal of cooperative intent (e.g. Hall et al., 2015; McCullough, Swartwout, Shaver, Carter, & Sosis, 2016; Power, 2017a, b; Purzycki & Arakchaa, 2013). Our results provide further evidence. However, they are also consistent with the hypotheses that costly behaviour serves to enhance cultural transmission or convince others of one's supernatural power. Costly religious behaviour may serve many functions simultaneously, or it may serve a single function and then have incidental effects in other domains.

Many scholars emphasize the costliness of prohibitions (e.g. Iannaccone, 1994; Soler, 2012; Sosis, 2006), but our research indicates that other factors might also influence which behaviours are prohibited. In line with the emphasis on costliness, we found that shamans are tabooed from sex and freely eating during healing ceremonies and initiations. However, we also found that, for permanent dietary taboos, cost seems tangential to whether an item is prohibited. Consider eels and flounders, both of which are prohibited to shamans. Eels ranked as the second most frequently consumed and favoured river species. The communities surveyed live along rivers and regularly fish in them, so this suggests a real, appreciable cost. In contrast, no participant named flounders when listing their preferred food items. This is not surprising. The Mentawai living in the interior of Siberut never come into contact with flounders, and in follow-up conversations, several participants admitted to never having eaten one.

Rather than only banning valuable food items, these permanent dietary taboos may also target peculiar animals. People regard atypical entities as impure or sacred (Douglas, 2002; Henrich & Henrich, 2010; Leach, 1964; Sperber, 1996), so rejecting them may enhance perceptions of a practitioner's difference. In fact, all of the foods tabooed to shamans are known as *makatai* (bad, broken, evil), and with the exception of the three-striped squirrel, all are anomalies. Gibbons are likened to humans (Schefold, 1972, 1982). The flounder is literally regarded as a split fish (*laitak katsila*). The white simakobu is the only white primate on the island. And eels are described as slippery and, in myths, are compared with snakes (Loeb, 1929a).

Readers should be aware of at least two limitations of our field experiment. First, the self-denying character engaged in voluntary self-denial beyond the typical restrictions observed by shamans. All shamans are expected to permanently reject eels, gibbons, and so on, as well as sex and uncontrolled eating during healing ceremonies, but the treatment character went beyond these restrictions, abstaining either from other hunted animals or completely from sex. Thus, our experiment risks capturing participants’ inferences about additional self-denial rather than inferences about adherence to typical prohibitions. Second, and relatedly, the experimental characters rejected items different from those normally forbidden to Mentawai shamans. Rather than rejecting eels, gibbons, and so on, the characters in the food-prohibition condition abandoned flying foxes, pangolins and Pagai Island macaques. How confident can we be that the inferences people made about these hypothetical forms of self-denial are equivalent to the inferences they make about normative abstention?

These points are important to consider, and future research should further probe how these variables bias people's inferences. Still, at least two lines of evidence suggest that our interpretation is justified. First, the inferences that participants made about a shaman who refrained from having sex seemed very similar to those they made about a shaman who denied himself hunted animals. Second, many participants spontaneously referred to the self-denying character as *makeikei* (tabooed), regardless of his prohibition. In doing so, they likened the novel forms of self-denial to other taboos, considering the shamans along a single dimension of prohibition. These two points suggest that participants’ inferences were not unique to the specific items but instead constituted more generalizable perceptions of people who self-deny. Nevertheless, the most conservative interpretation is that Mentawai participants infer cooperativeness, credibility and supernatural power from shamans who self-deny and that those inferences seem similar regardless of whether shamans refrain from sex or from eating large, hunted game.

In sum, we have provided evidence that religious leaders in a small-scale society observe permanent and periodic prohibitions and, in doing so, garner perceptions of belief, cooperativeness and supernatural powers. This project helps elucidate why figures across time and space, from the shamans of rainforest horticulturalists to the memorialized prophets of the world's major religions, have rejected sex, food and social contact on their journeys towards leadership and apparent divinity.

## Data Availability

The data and code for this study are available and can be found at the project OSF site: https://osf.io/3mbkz

## Supplementary material

For supplementary material accompanying this paper visit http://dx.doi.org/10.1017/ehs.2020.32. click here to view supplementary material
