# Supplementary material for: Why do religious leaders observe costly prohibitions? Examining taboos on Mentawai shamans
Source: Evol Hum Sci. 2020 Jun 11;2:e32. doi: 10.1017/ehs.2020.32 (PMC10427447; doi:10.1017/ehs.2020.32)
Supplement: Supplementary file 1 [file S2513843X20000328sup001.pdf]

# Why do religious leaders observe costly prohibitions?
## Examining taboos on Mentawai shamans

## SUPPLEMENTARY MATERIAL

Manvir Singh and Joseph Henrich
*Department of Human Evolutionary Biology, Harvard University, Cambridge, MA 02138*

6 June 2020

---

## Supplementary Methods and Materials

All data and R code are available at https://osf.io/3mbkz.

### Questions

Question IDs (e.g., beli1, coop1) match those in the dataset. Questions that did not load onto the relevant latent factor are marked with an asterisk (*).

**Belief**
beli1: *For example, there is a ceremony. Of the two of them here, someone eats sour* [the consumption of sour is tabooed during ceremonies and believed to cause misfortune]. *Who is it?*
beli2: *According to you, who believes more in* Arat Sabulungan [Mentawai religion]*?*
beli3: *According to you, who follows taboos less?*

**Cooperativeness**[1]
coop1: *According to you, who is a thief?*
coop2: *According to you, who shares meat more?*
*coop3: *For example, there is a burning house. Who goes to help?*
*trus1: *For example, you are not here because you are working or with family somewhere far. Who do you look for to help take care of your children here?*
trus2: *For example, you need personal advice on a family issue. Who do you ask?*

[1] These questions were originally designed to target distinct inferences: a general cooperative disposition (is the shaman cooperative towards others?) and trustworthiness (would the participant trust the shaman in particular?). But given the similarity between these inferences and that they address the same prediction, we tested whether there is greater internal reliability among the questions as a single construct. We found evidence that there was. Cronbach's alpha was higher for all questions as a single construct than for two separate constructs. As described in the main text, we also found that coop1, coop2, and trus2 loaded onto a single construct whereas coop3 and trus1 did not (each instead loaded on its own unique factor).

**Difference**
*diff1: *Whose thoughts are closer to those of a non-shaman?*
diff2: *Whose thoughts are farther from those of a non-shaman?*
diff3: *Whose body is closer to that of a non-shaman?*

**Power**
powe1: *Who has weaker medicine?*
powe2: *Who has stronger medicine?*
powe3: *Who has stronger magic?*

## Supplementary Figures

**Figure S1.** The frequency with which participants (N = 68) selected the non-self-denying (0) or self-denying (1) character as exhibiting the investigated trait (responses to reverse-worded questions have been inverted). Questions that did not load onto the latent structure are marked with an asterisk (*). See Table S6 for raw counts.

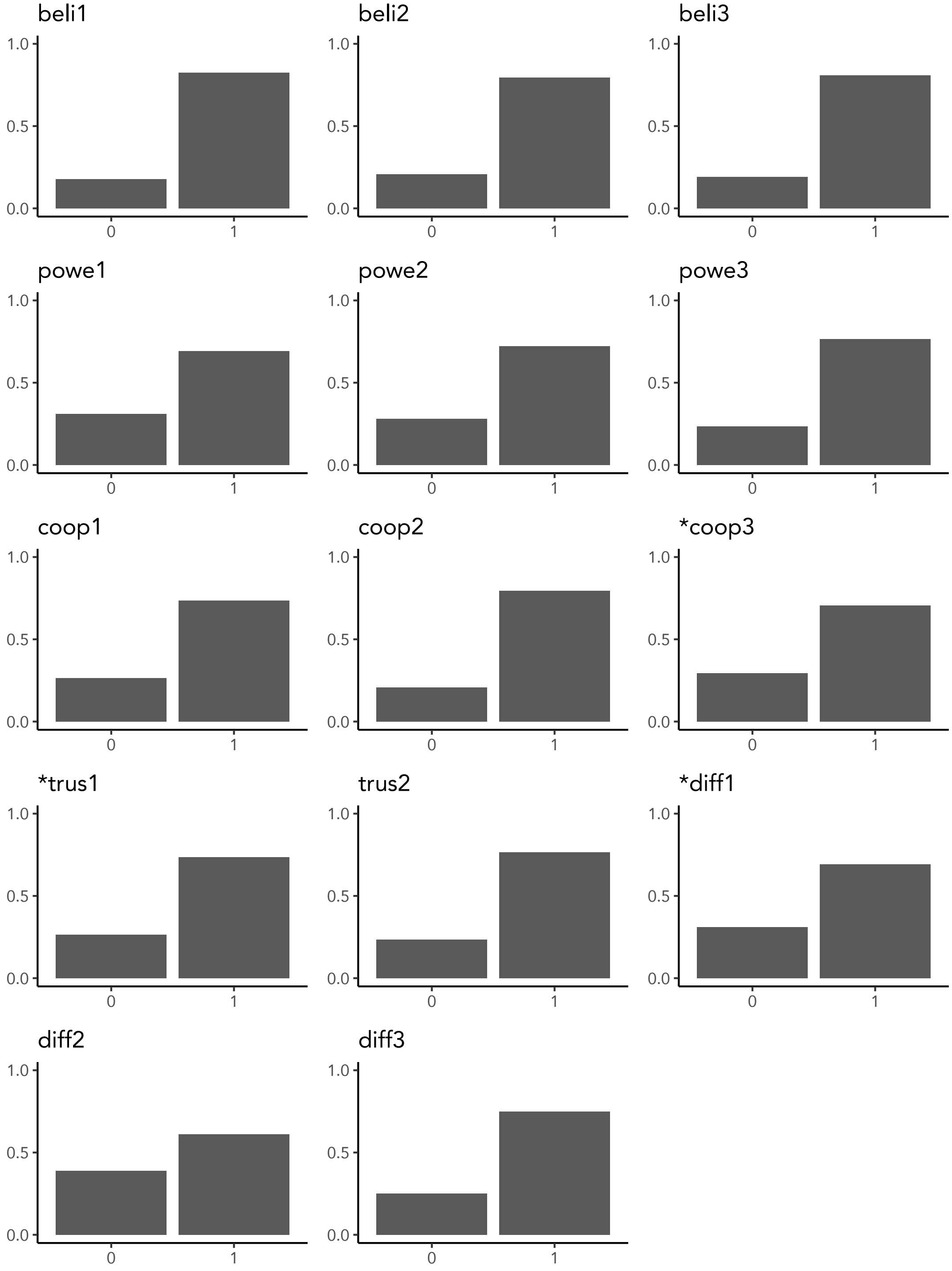

**Figure S2.** A histogram showing the frequency with which participants chose the self-denying shaman as exhibiting the trait of interest (belief, cooperativeness, difference, power). Fifteen participants chose the self-denying shaman for all 14 questions (this includes, for the reverse-coded questions, selecting the non-self-denying shaman). Four participants never chose the self-denying shaman (again, this includes selecting the self-denying shaman for reverse-coded questions).

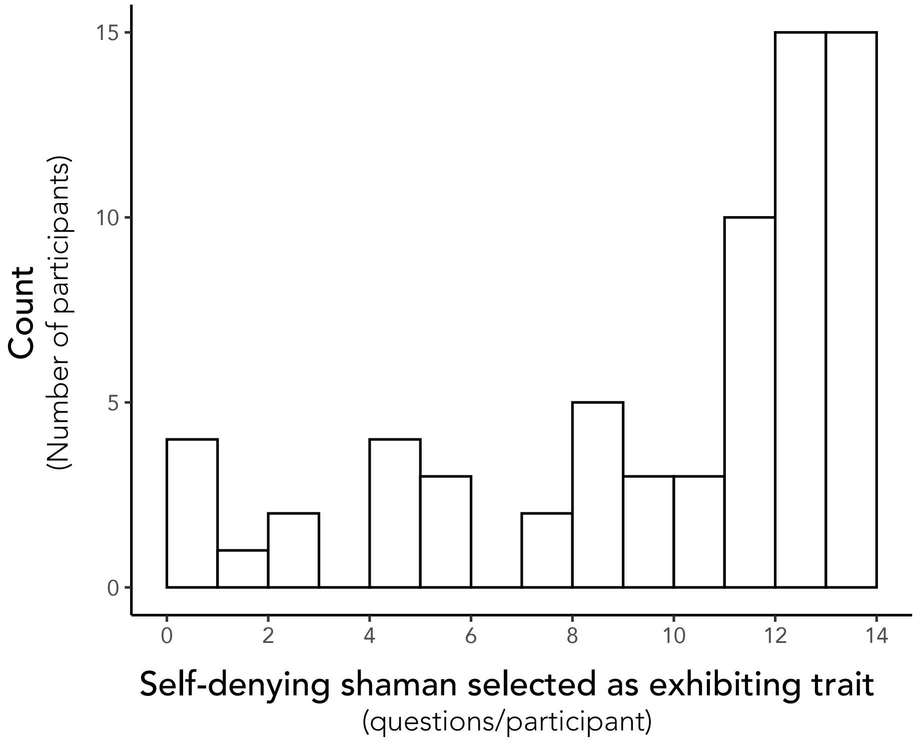

## Supplementary Tables

**Table S1.** Raw frequencies with which respondents named taboos on shamans during initiation and healing across four cultural regions. See Fig. 3 and the main text for details.

| | | FREE-LIST RESPONSES | | | | | | | | | | | | | | AGGREGATED | | | | |
|---|---|---|---|---|---|---|---|---|---|---|---|---|---|---|---|---|---|---|---|---|
| | | Clear brush for gardening | Cook | Cut/break | Display anger or yell | Eat fern | Eat sour | Eat without self-control | Fast intermittently | Have sex (adultery) | Have sex (spouse) | Have sex (general) | Plant | Shower | Work (general) | Cooking/work | Disturb household | Eat | Groom | Have sex |
| INITIATION | SAB | 0.00 | 0.00 | 0.15 | 0.05 | 0.15 | 0.25 | 0.40 | 0.00 | 0.20 | 0.20 | 0.30 | 0.00 | 0.00 | 0.10 | 0.20 | 0.05 | 0.65 | 0.00 | 0.55 |
| | SAR | 0.12 | 0.04 | 0.04 | 0.04 | 0.12 | 0.27 | 0.15 | 0.23 | 0.12 | 0.19 | 0.65 | 0.12 | 0.12 | 0.19 | 0.42 | 0.04 | 0.65 | 0.15 | 0.88 |
| | SIL | 0.14 | 0.05 | 0.05 | 0.24 | 0.00 | 0.38 | 0.10 | 0.05 | 0.19 | 0.29 | 0.33 | 0.00 | 0.14 | 0.29 | 0.33 | 0.24 | 0.43 | 0.14 | 0.57 |
| | TAI | 0.05 | 0.05 | 0.00 | 0.05 | 0.05 | 0.20 | 0.30 | 0.15 | 0.25 | 0.25 | 0.20 | 0.25 | 0.05 | 0.35 | 0.45 | 0.05 | 0.70 | 0.10 | 0.55 |
| HEALING | SAB | 0.05 | 0.00 | 0.05 | 0.15 | 0.05 | 0.10 | 0.40 | 0.00 | 0.05 | 0.15 | 0.35 | 0.05 | 0.10 | 0.05 | 0.15 | 0.15 | 0.50 | 0.10 | 0.45 |
| | SRR | 0.19 | 0.04 | 0.08 | 0.08 | 0.15 | 0.12 | 0.15 | 0.00 | 0.19 | 0.08 | 0.38 | 0.04 | 0.00 | 0.15 | 0.46 | 0.12 | 0.50 | 0.00 | 0.65 |
| | SIL | 0.24 | 0.00 | 0.00 | 0.29 | 0.00 | 0.10 | 0.05 | 0.00 | 0.14 | 0.29 | 0.24 | 0.05 | 0.00 | 0.48 | 0.67 | 0.29 | 0.19 | 0.10 | 0.57 |
| | TAI | 0.15 | 0.00 | 0.10 | 0.05 | 0.00 | 0.05 | 0.15 | 0.05 | 0.15 | 0.20 | 0.45 | 0.15 | 0.00 | 0.15 | 0.35 | 0.05 | 0.30 | 0.00 | 0.75 |

**Table S2.** Raw frequencies with which respondents reported dietary taboos on shamans across four cultural regions. See Fig. 4 and the main text for details.

| | Eel *Anguilla bicolor* | Fern *Diplazium esculentum* | Flounder Pleuronectiformes | Gibbon *Hylobates klossii* | Green broadbill *Calyptomena viridis* | Mentawai squirrel *Callosciurus melanogaster* | Mushroom Unknown poliphore | Palm hearts {e.g., sago, coconut} | Simakobu (white morph) *Simias concolor* | Taro leaves *Colocasia esculenta* | Three-striped squirrel *Lariscus obscurus* | Turmeric *Cucuma longa* | Turtle *Heosemys spinosa* | Mentawai Langur* *Potenziani presbytis* |
|---|---|---|---|---|---|---|---|---|---|---|---|---|---|---|
| SAB | 1.00 | 0.89 | 0.95 | 0.89 | 0.16 | 0.68 | 0.42 | 1.00 | 1.00 | 0.58 | 0.84 | 0.74 | 0.79 | 0.00 |
| SAR | 1.00 | 0.27 | 0.92 | 0.85 | 0.62 | 0.15 | 0.08 | 0.31 | 0.96 | 0.19 | 0.96 | 0.04 | 0.23 | 0.00 |
| SIL | 1.00 | 1.00 | 0.86 | 1.00 | 0.48 | 1.00 | 0.19 | 1.00 | 0.95 | 0.24 | 1.00 | 0.10 | 1.00 | 0.05 |
| TAI | 0.95 | 1.00 | 0.90 | 1.00 | 0.35 | 0.95 | 0.90 | 1.00 | 0.95 | 1.00 | 1.00 | 0.90 | 0.95 | 0.00 |

**Table S3.** Probabilities that different items are tabooed in the four cultural regions according to cultural consensus analyses.

| | Eel *Anguilla bicolor* | Fern *Diplazium esculentum* | Flounder Pleuronectiformes | Gibbon *Hylobates klossii* | Green broadbill *Calyptomena viridis* | Mentawai squirrel *Callosciurus melanogaster* | Mushroom Unknown poliphore | Palm hearts {e.g., sago, coconut} | Simakobu (white morph) *Simias concolor* | Taro leaves *Colocasia esculenta* | Three-striped squirrel *Lariscus obscurus* | Turmeric *Cucuma longa* | Turtle *Heosemys spinosa* | Mentawai langur* *Potenziani presbytis* |
|---|---|---|---|---|---|---|---|---|---|---|---|---|---|---|
| SAB | >0.99 | >0.99 | >0.99 | >0.99 | <0.01 | >0.99 | 0.47 | >0.99 | >0.99 | >0.99 | >0.99 | >0.99 | >0.99 | <0.01 |
| SAR | >0.99 | <0.01 | >0.99 | >0.99 | >0.99 | <0.01 | <0.01 | <0.01 | >0.99 | <0.01 | >0.99 | <0.01 | <0.01 | <0.01 |
| SIL | >0.99 | >0.99 | >0.99 | >0.99 | 0.77 | >0.99 | <0.01 | >0.99 | >0.99 | <0.01 | >0.99 | <0.01 | >0.99 | <0.01 |
| TAI | >0.99 | >0.99 | >0.99 | >0.99 | <0.01 | >0.99 | 1.00 | >0.99 | >0.99 | >0.99 | >0.99 | >0.99 | >0.99 | <0.01 |

**Table S4.** Details about cultural consensus analyses. According to Weller (2007), a consensus model can be used to represent a group's responses when the Comrey's ratio approximates 3 to 1 or greater. The results show that the model can used for each of the four cultural regions but not when combining participants' responses from across cultural regions into a single set. This is to be expected given that the combined dataset mixes participants from different cultural communities.

| | Negative competencies | Competencies over 1 | Comrey's Ratio |
|---|---|---|---|
| SAB | 0 | 0 | 2.67 |
| SAR | 1 | 0 | 2.90 |
| SIL | 0 | 0 | 3.73 |
| TAI | 0 | 0 | 3.63 |
| All rivers combined | 0 | 0 | 1.66 |

**Table S5.** The twenty-four nonaquatic, foraged animals presented to subjects. Items tabooed to shamans in all four regions are shaded. See also Fig. 4.

| Specific name | English name | Mentawai name | Mean ranking | Standard deviation |
|---|---|---|---|---|
| *Anthracoceros coronatus* | Malabar pied hornbill | *kailaba* | 6.46 | 1.39 |
| *Ardea sumatrana* | Great-billed heron | *meccau* | 2.28 | 1.32 |
| *Calyptomena viridis* | Green broadbill | *luikluik* | 4.59 | 1.76 |
| *Centropus sinensis* | Greater coucal | *kemut* | 5.13 | 1.59 |
| *Ceyx erithacus* | Three-toed kingfisher | *sikoplaitak* | 1.28 | 0.60 |
| *Cuculux fugax* | Hawk-cuckoo | *buccik* | 4.28 | 1.39 |
| *Cuora amboinensis* | Box turtle | *lokipat* | 3.10 | 1.63 |
| *Dicaeum cruentataum* | Scarlet-backed flowerpecker | *ritdit* | 3.73 | 1.74 |
| *Dicaeum trigonistigma* | Orange-bellied flowerpecker | *dhadhatdhat* | 4.36 | 2.05 |
| *Geomyda spinosa* | Spiny turtle | *toulu* | 3.70 | 1.71 |
| *Hylarana nicobariensis* | Nicobar golden-backed frog | *loloakkek* | 4.05 | 1.88 |
| *Hylobates klossii* | Kloss's gibbon | *bilou* | 4.93 | 1.95 |
| *Lariscus obscurus* | Three-striped squirrel | *soksak* | 2.50 | 1.85 |
| *Macaca pagensis* | Pagai macaque | *obaketa* | 5.88 | 1.81 |
| *Occidozyga laevis* | Puddle frog | *utetsopak* | 6.63 | 1.41 |
| *Paradoxurus hermaphroditus siberu* | Siberut palm civet | *lamusek* | 3.45 | 2.18 |
| *Phaenicophaeus curvirostris* | Chestnut-breasted malkoha | *koitkot* | 5.83 | 1.41 |
| *Pycnonotus melanoleucos* | Black-and-white bulbul | *pusitattat* | 4.59 | 1.87 |
| *Pycnonotus plumosus* | Olive-winged bulbul | *rotdot* | 5.56 | 1.70 |
| *Rhynchophorus ferrugineus* | Sago palm weevil (larvae) | *tamara* | 6.40 | 2.37 |
| *Simias concolor* | Simakobu monkey (black morph) | *simakobuk* | 6.90 | 1.37 |
| *Simias concolor* | Simakobu monkey (white morph) | *simabulau* | 3.90 | 1.57 |
| *Sturnus sturninus* | Daurian starling | *ngorut* | 6.88 | 1.38 |
| *[Tadpole]* | Tadpole | *kalabbok* | 1.48 | 0.75 |

**Table S6.** How often participants (N = 68) selected the non-self-denying (0) or self-denying (1) character as exhibiting the investigated trait (responses to reverse-coded questions have been inverted). Questions that did not load onto the relevant latent structure are marked with an asterisk (*).

| Question | Response | Count | Frequency |
|---|---|---|---|
| beli1 | 1 | 56 | 0.824 |
| beli1 | 0 | 12 | 0.176 |
| beli2 | 1 | 54 | 0.794 |
| beli2 | 0 | 14 | 0.206 |
| beli3 | 1 | 55 | 0.809 |
| beli3 | 0 | 13 | 0.191 |
| powe1 | 1 | 47 | 0.691 |
| powe1 | 0 | 21 | 0.309 |
| powe2 | 1 | 49 | 0.721 |
| powe2 | 0 | 19 | 0.279 |
| powe3 | 1 | 52 | 0.765 |
| powe3 | 0 | 16 | 0.235 |
| coop1 | 1 | 50 | 0.735 |
| coop1 | 0 | 18 | 0.265 |
| coop2 | 1 | 54 | 0.794 |
| coop2 | 0 | 14 | 0.206 |
| *coop3 | 1 | 48 | 0.706 |
| *coop3 | 0 | 20 | 0.294 |
| *trus1 | 1 | 50 | 0.735 |
| *trus1 | 0 | 18 | 0.265 |
| trus2 | 1 | 52 | 0.765 |
| trus2 | 0 | 16 | 0.235 |
| *diff1 | 1 | 47 | 0.691 |
| *diff1 | 0 | 21 | 0.309 |
| diff2 | 1 | 41 | 0.612 |
| diff2 | 0 | 26 | 0.388 |
| diff3 | 1 | 51 | 0.750 |
| diff3 | 0 | 17 | 0.250 |

**Table S7.** Loadings from exploratory factor analyses assessing whether each of the four sets of questions load onto a single construct. The dotted lines separate different factor analyses.

| Question | MR1 | MR2 | MR3 |
|---|---|---|---|
| beli1 | 0.73 | 0.08 | - |
| beli2 | 0.93 | -0.06 | - |
| beli3 | 0.96 | 0.02 | - |
| powe1 | 0.97 | -0.11 | - |
| powe2 | 0.99 | 0.03 | - |
| powe3 | 0.75 | 0.18 | - |
| coop1 | 0.99 | -0.13 | 0.10 |
| coop2 | 0.90 | 0.09 | -0.05 |
| coop3 | 0.01 | 0.93 | 0.09 |
| trus1 | 0.02 | 0.05 | 0.96 |
| trus2 | 0.66 | 0.29 | -0.07 |
| diff1 | 0.00 | 0.68 | - |
| diff2 | 0.77 | -0.08 | - |
| diff3 | 0.57 | 0.41 | - |

**Table S8.** Loadings from exploratory factor analysis conducted with responses to all questions. The factor analysis does not produce four distinct factors corresponding with each of the four sets of questions, indicating correlations among responses across questions.

| Question | MR1 | MR2 | MR3 | MR4 |
|---|---|---|---|---|
| beli1 | 0.53 | 0.22 | 0.45 | 0.28 |
| beli2 | 0.68 | 0.52 | 0.40 | 0.17 |
| beli3 | 0.58 | 0.55 | 0.39 | - |
| powe1 | 0.80 | 0.42 | 0.21 | 0.10 |
| powe2 | 0.90 | 0.36 | 0.23 | - |
| powe3 | 0.57 | 0.43 | 0.36 | 0.12 |
| coop1 | 0.44 | 0.80 | 0.11 | 0.14 |
| coop2 | 0.28 | 0.87 | 0.21 | 0.14 |
| coop3 | 0.17 | 0.23 | 0.91 | - |
| trus1 | 0.69 | 0.20 | 0.62 | 0.27 |
| trus2 | 0.15 | 0.66 | 0.34 | 0.35 |
| diff1 | 0.70 | 0.11 | - | 0.15 |
| diff2 | 0.17 | 0.21 | 0.13 | 0.95 |
| diff3 | 0.38 | 0.36 | 0.55 | 0.40 |

**Table S9.** Indices of internal reliability and unidimensionality for the four sets of questions. The table includes values both when including (I) and excluding (E) questions that did not load onto the relevant factor.

| | Cronbach's alpha | Average inter-item correlation | Unidimensional criterion[1] |
|---|---|---|---|
| Belief | 0.77 | 0.52 | 1 |
| Power | 0.81 | 0.58 | 1 |
| Cooperativeness (I) | 0.76 | 0.38 | 0.99 |
| Cooperativeness (E) | 0.76 | 0.51 | 1 |
| Difference (I) | 0.57 | 0.31 | 1 |
| Difference (E) | 0.55 | 0.38 | 1 |

[1]The unidimensional criterion is a recent measure of unidimensionality available using the unidim function in the psych package in R (Revelle 2019).

**Table S10.** Comparison of estimated probabilities with and without data exclusion; 95% CIs are included in brackets.

| | Excluding participants | Without excluding participants |
|---|---|---|
| Belief | 0.92<br>[0.84, 0.96] | 0.84<br>[0.76, 0.90] |
| Cooperativeness | 0.88<br>[0.78, 0.94] | 0.82<br>[0.73, 0.89] |
| Difference | 0.78<br>[0.64, 0.88] | 0.73<br>[0.61, 0.83] |
| Power | 0.84<br>[0.72, 0.91] | 0.77<br>[0.66, 0.85] |

**Table S11.** Comparison of estimated probabilities with and the without removal of questions; 95% CIs are included in brackets.

| | With removal of coop3, trus1, diff1 | Without removal |
|---|---|---|
| Belief | 0.92<br>[0.84, 0.96] | 0.91<br>[0.84, 0.95] |
| Cooperativeness | 0.88<br>[0.78, 0.94] | 0.85<br>[0.76, 0.91] |
| Difference | 0.78<br>[0.64, 0.88] | 0.77<br>[0.65, 0.86] |
| Power | 0.84<br>[0.72, 0.91] | 0.83<br>[0.72, 0.90] |

**Table S12.** Results of the logistic regression (observations = 747; participants = 68). The outcome is a binary variable representing whether or not the participant chose the self-denying shaman for a given question.

| | Estimate | SE | z |
|---|---|---|---|
| Intercept | 1.35 | 0.73 | 1.84 |
| Trait[1] | | | |
| *Cooperativeness* | -0.44 | 0.31 | -1.40 |
| *Difference* | -1.16 | 0.34 | -3.15 |
| *Power* | -0.79 | 0.31 | -2.54 |
| Sex[1] | 0.71 | 0.68 | 1.04 |
| Stimuli counterbalance[2] | 0.26 | 0.59 | 0.44 |
| Category of self-denial[1] | 0.92 | 0.61 | 1.52 |

Mixed effects logistic regression with random effects for participant, conducted with the glmer function (lme4 package) in R. The effects package was used to produce the probability estimates presented in the text and in Fig. 5.
[1]Reference levels are Belief (Trait), Male (Sex), Food (Category of self-denial).
[2]Stimuli counterbalance is a dummy variable referring to whether the self-denying shaman had one set of counter-balanced text or the other.

**Table S13.** Estimated odds ratios for all possible pairs of trait inferences.

| | Odds ratio | SE | z | Adjusted p |
|---|---|---|---|---|
| Belief-Cooperativeness | 1.55 | 0.49 | 1.40 | 0.486 |
| Belief-Difference | 3.19 | 1.08 | 3.42 | 0.004 |
| Belief-Power | 2.20 | 0.68 | 2.54 | 0.055 |
| Cooperativeness-Difference | 2.05 | 0.67 | 2.21 | 0.108 |
| Cooperativeness-Power | 1.42 | 0.42 | 1.18 | 0.486 |
| Difference-Power | 0.69 | 0.22 | -1.17 | 0.486 |

# References

Revelle, W. (2019) psych: Procedures for psychological, psychometric, and personality research. R package version 1.19.12. https://cran.r-project.org/package=psych

Weller, S. C. (2007) Cultural consensus theory: Applications and frequently asked questions. *Field Methods* 19:339–368. doi:10.1177/1525822X07303502
